# Beyond Acephalic Spermatozoa: The Complexity of Intracytoplasmic Sperm Injection Outcomes

**DOI:** 10.1155/2020/6279795

**Published:** 2020-02-10

**Authors:** Hua Nie, Yunge Tang, Weibing Qin

**Affiliations:** ^1^NHC Key Laboratory of Male Reproduction and Genetics, Guangzhou, China; ^2^Department of Central Laboratory of Family Planning Research Institute of Guangdong Province of China, Guangzhou, China; ^3^Department of Central Laboratory of Family Planning Special Hospital of Guangdong Province of China, Guangzhou, China

## Abstract

This review analyses the genetic mechanisms of acephalic spermatozoa (AS) defects, which are associated with primary infertility in men. Several target genes of headless sperms have been identified but intracytoplasmic sperm injection (ICSI) outcomes are complex. Based on electron microscopic observations, broken points of the sperm neck are AS defects that are based on various genes that can be classified into three subtypes: *HOOK1*, *SUN5*, and *PMFBP1* genes of subtype II; *TSGA10* and *BRDT* genes of subgroup III, while the genetic mechanism(s) and aetiology of AS defects of subtype I have not been described and remain to be explored. Interestingly, all AS sperm of subtype II achieved better ICSI outcomes than other subtypes, resulting in clinical pregnancies and live births. For subtype III, the failure of clinical pregnancy can be explained by the defects of paternal centrioles that arrest embryonic development; for subtype I, this was due to a lack of a distal centriole. Consequently, the embryo quality and potential ICSI results of AS defects can be predicted by the subtypes of AS defects. However, this conclusion with regard to ICSI outcomes based on subtypes still needs further research, while the existence of quality of oocyte and implantation failure in women cannot be ignored.

## 1. Introduction

Of all infertility diseases in men, having acephalic spermatozoa (AS; OMIM: 617187) is one of the most serious male spermatogenic disorders that yields a unique abnormal sperm morphology: a large number of decapitated and decaudated spermatozoa with a small number of abnormal head-tail junction sperm in ejaculates [1, 2]. Based on the World Health Organization [3] guidelines, the lower reference value for normal sperm is set at 4%. This means that not all AS affected individuals are infertile. Separated heads and tails of sperm are commonly observed in both fertile and infertile population groups [4]. The limitation of specific management and a lack of knowledge of the pathogenesis make the clinical prognosis of AS defects unpredictable as these usually do not respond to pharmacological intervention [4–6]. Similar conditions can be observed within consanguinities and unrelated patients [4]. The ratio of headless to tailless sperm in semen is over 30 : 1 [7] since most of the heads are phagocytosed by Sertoli cells [4, 8, 9].

The aetiology and pathogenesis of AS affected individuals have been shown to be genetic in origin, with five identified AS genes [10–12]: *SUN5*, *BRDT*, *PMFBP1*, *TSGA10*, and *HOOK1*. The natural fertilising abilities of these genes are lost because almost all AS sperm are broken in two. Intracytoplasmic sperm injection (ICSI) is the only technique so far in which abnormal or immotile sperm have the opportunity to fertilise oocytes successfully [13, 14]. Meanwhile, Sha et al. [11] believed that ICSI was the only solution to AS defects; however, not all AS-deficient sperm achieved the same clinical results [15].

Schatten [16] highlighted the key role of the sperm centrosome in the ability of sperm to be fertile and in the early development of zygotes. While the studies of Rawe et al. [17] indicated that paternal centriolar dysfunction can arrest the development of the embryo. Thus, this review has focused on the genetic contributions of AS and their possible prognosis, with an in-depth discussion on whether the AS sperm subtypes can predict the complexity of outcomes of ICSI.

## 2. Candidate Genes of AS Defects Can Be Classified Based on Ultrastructural Observations

**Figure 1:**
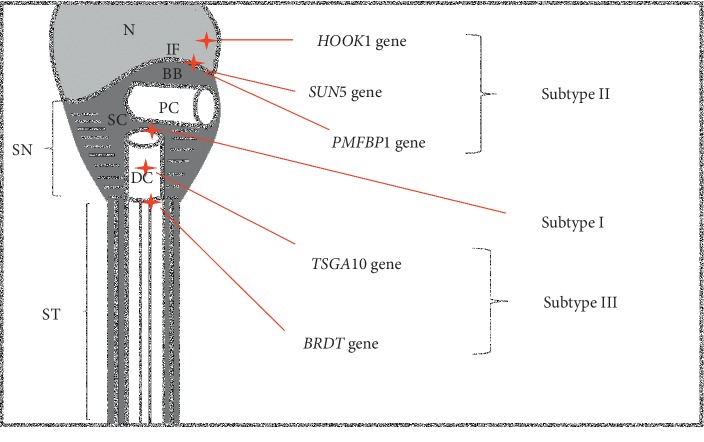
Premature module of sperm with locations of AS-related genes and subtypes. Red star markers represent the location of each AS gene in the sperm. Meanwhile, based on their locations, these AS-related genes can be grouped into three subtypes: I, II, and III. N: nucleus; PC: proximal centriole; DC: distal centriole; SC: segmented columns; ST: sperm tail; SN: sperm neck (connecting piece); BB: basal body; IF: implantation fossa.

The unique structure of the sperm neck includes the implantation fossa, basal body, centrosome, and segmented columns (shown in Figure 1). The sperm centrosome contains two perpendicular centrioles: proximal and distal centrioles. The proximal centriole moves into the implantation fossa (a concavity located at the distal end of the nuclear envelope) and attaches to the caudal end of the nucleus, inducing the formation of a basal body, while the distal centriole can be used as a template for tail growth [4, 8, 18–21]. The implantation fossa and centriole-lagellar complex connect to each other to form a head-tail articulation (sperm neck) [4], which is very stable and allows sperm to move forwards. Generally, each of these components of the sperm neck is missing, leading to a fragile head-tail articulation, and the headless sperms are formed once the head-tail articulation is separated.

The formation of AS likely has its origin during spermiogenesis. It may be initiated in round haploid germ cells involving thousands of different gene-coding proteins [22]. Interestingly, neither the implantation fossa nor the basal body of sperm is formed in most AS cases [23]. The size and morphology of the decaudated head in some sperm are similar to that of normal sperm, with a 1/3 ratio of acrosomes to heads [12]. This suggests AS defects may be a result of a fragile head-tail connection [6, 8] or because of abnormal attachments between centrosomes and nuclei [7]. The head and tail of sperm remain independent of each other until they mature. If the head and tail are not on the same axis during spermatogenesis, the fragility of the head-neck junction may be increased. Meanwhile, aplasia may also be the cause of the separation of the heads and tails of sperm because of an abnormal structure of the implantation fossa and/or the basal body [7]. Soley [24] believed that abnormal locations of the vacuoles and incomplete mitochondria can also physically prevent the formation of a normal head-neck junction. The droplets attached to the proximal site of the sperm tail can be misrecognised as oligozoospermia or pinhead-like sperm in some cases [25].

Based on ultrastructural observations and genetic variations, the broken points of sperm necks are AS defects that can be classified into three subtypes (shown in Table 1): I, II, and III. The differences in AS phenotypes between subtypes strongly highlight the genetic variations found in AS defects, which have been reported in both animal and human studies [4, 10, 11, 27].

### 2.1. AS Subtype I Defect

The breakage in this subtype is located between the two centrioles (Table 1 and Figure 1). A significant separation of the two centrioles can be identified by electron microscopy: the tailless head connects to the complete proximal centriole [6, 20], with an intact implantation fossa and basal body [20, 23, 28]. This means that the separated head and tail, respectively, contain their own complete structures. Although distal and proximal centrioles are separated during a very early spermatid stage, the distal centriole alone is enough to grow a complete flagellum, keeping progressive motility in the ejaculate [20, 23, 28]. However, only this type of AS defect has been observed to date, the genetic mechanism and aetiology of which remain to be explored.

### 2.2. AS Defects Subtype II and Related Genes

The separation in subtype II is located between the nucleus and proximal centriole: the lack of an implantation fossa and/or basal body in the tailless head, and the presence of intact proximal and distal centrioles in the headless tail have been reported [2, 10, 12, 27, 29]. In the tailless head, an intact nucleus, with/without a typical acrosome structure, as well as a compressed nucleus can be observed, but the basal body is generally absent as shown in Table 1. A cytoplasmic droplet sometimes can be observed in the proximal end of a headless sperm tail, which is often mistaken for a pink-like head [7]. The normal arranged proximal centriole, however, is usually contained in a tailless head with segmented columns [12]. Additionally, Perotti et al. [30] described how a headless tail contains a proximal centriole, mitochondrial sheath, and intact tail structure, localising to the correct region in all cases of this subtype. Therefore, the absence or aplasia of a basal body and implantation fossa seems to be the major cause of subtype II AS defects. A lack of implantation fossa can inhibit normal contact with the centrosome and lead to a failure to induce the formation of a basal body [6, 29, 30]. Mutated *SUN5* and *HOOK1* genes associated with AS defects show similar phenotypes, which match subtype II [2, 12]. The headless tails from this subtype of AS can still move forwards in ejaculated semen [12]. Additionally, based on observations, the tails of headless sperm with mutated *PMFBP1* gene-related AS defects contain proximal and distal centrioles [10].

Genetic defects related to the SAD1 and UNC84 domain containing 5 (*SUN5*) gene, which is the first identified target gene of an AS defect [10], have been reported in both animal models and human studies [27]. SUN5 is a transmembrane protein located on the nuclear envelope facing the basal body [11, 31, 32]. Shang et al. [27] believed that the function of the SUN5 protein is to promote binding and interaction between the implantation fossa and basal body, ensuring that the head is anchored to the tail of the sperm. More than 10 mutations of the *SUN5* gene associated with AS have been reported, including missense, nonsense, splicing variant, and frameshift [27]. As shown in Table 2, six of these are missense variations with an unknown effect on the SUN5 protein. Shang et al. [27] suggested that each of these mutations, located in the coiled-coil and SUN domains, may impair the secondary structure of the SUN protein, leading to separation of the head and neck. It has been highlighted that c.340G > A variation in the *SUN5* gene may affect the intron splicing site [10]; however, experimental studies are lacking. In addition to intronic splicing, genetic variations have been found that result in the disruption of the SUN5 protein. Both nonsense and frameshift variations result in truncated SUN5 proteins, the functions of which were lost [27]. Meanwhile, a mutated *SUN5* gene can reduce the expression of outer dense fiber 1 (ODF1) protein, which may be the cause of the misarranged structure of the mitochondrial sheath [35]. Thus, homozygous and/or compound heterozygous variations of the *SUN5* gene can result in the formation of primary AS defects.

According to the work of Sha et al. [11], polyamine modulated factor 1 binding protein 1 (*PMFBP1*) is another candidate gene for AS defects. Immunofluorescence staining in an animal model suggested that the PMFBP1 protein is specifically expressed in both the implantation fossa and basal body regions of spermatozoa [11]. The sandwiched localisation of the PMFBP1 protein in the central region between SUN5 and TSGA10 proteins has been found to connect a portion of the neck to the head and tail of sperm, which is used as a scaffold protein in the attachment of the basal body to the nuclear membrane [10, 11]. However, a further study suggested that these three proteins failed to interact with each other, leading to the formation of acephalic sperm [11]. PMFBP1 protein is found in residual droplets only at a distal site of the neck, but not in the implantation fossa as SUN5. This indicates that PMFBP1 is a downstream protein of the SUN5 protein that helps the SUN5 protein to bring the heads and tails of sperm together [10, 11]. It is believed that nearly half of all defects of AS sperm are caused by *SUN5* and *PMFBP1* genes in the Chinese population [10]. Mutation-associated AS infertility of the *PMFBP1* gene has been reported in both related and unrelated individuals with AS defects [10]. Homozygous or compound heterozygous nonsense and frameshift variations of *PMFBP1* genes that lead to a loss of function of the protein (Table 2) have been found in AS affected patients.

In addition, HOOK1 is a known manchette-bound protein that belongs to the family of HOOK proteins. Manchette is a unique structure of microtubules occurring during the development of spermatids, based on a review by Chen et al. [36], the function of which is to ensure that the sperm nucleus elongates and the sperm tail condenses. It is believed that the manchette bridges proteins transportation between the head and tail of sperm and promotes protein redistribution [12]. The study of Mendoza-Lujambio et al. [37] highlighted how the *HOOK1* gene was nonfunctional in sperm head morphology but was a candidate gene in the response to acephalic spermatozoa syndrome [12]. The function of the HOOK1 protein is to regulate the attachment between the centrosome and nuclear envelope during spermatogenesis. A mutated *HOOK1* gene and dysfunctional HOOK1 protein results in a malfunction of the migration of the centrosome: the head and the tail of sperm are not on the same axis during spermatogenesis, consequently resulting in the formation of headless sperm with an altered implantation fossa and basal body [12, 38]. Thus, genetic variation in HOOK1 is not only associated with intramanchette transport, but also the head-tail coupling apparatus [12]. A homozygous nonsense variation in the *HOOK1* gene affecting the coiled-coil domain of the HOOK1 protein has been identified in pathogenic effects in spermatogenesis [12]. This means that the mutated *HOOK1* gene is crucial to the formation of headless sperm.

### 2.3. AS Defects Subtype III and Related Genes

The broken point of subtype III is sited between the distal centriole and midpiece as outlined in Table 1. Cytoplasmic droplets, with/without the incomplete mitochondrial sheath, can be observed in the proximal end of the tail, which sometimes are misrecognised as globozoospermia [7]. As observed by Li et al. [26], in AS defective sperm associated with a mutated *BRDT* gene, the whole mitochondrial sheath is missing from the midpiece of the headless sperm tail. Similarly, an impaired mitochondrial sheath can also be found for the mutated *TSGA10* gene in AS defect cases [34].

TSGA10 protein is one of the scaffold proteins of the centrosome that can accumulate in the distal centriole and midpiece of sperm. It is connected to the PMFBP1 protein upwards and ODF2 protein downwards [34]. A homozygous frameshift of the *TSGA10* gene causes a truncated TSGA10 protein that may affect the elongation of the sperm tail and disrupt the formation of the sperm midpiece [34]. Western blot analysis indicated that the function of the truncated protein was lost, and distal centriole and midpiece structures of sperm were impaired. As Sha et al. [34] described, only a partial mitochondrial sheath can be observed from the headless tail, which suggested that the breakage of TSGA10 associated with AS sperms is located between the distal centriole and the midpiece.

The BRDT protein, in comparison, is a member of the bromodomain and extraterminal subfamily and regulates meiosis in spermatocytes [39], the transcription of gene expression [40], and the elongation of sperm tails during spermatogenesis [41]. A loss of function of the mutated *BRDT* gene is associated with severe oligozoospermia and/or azoospermia disorders in western countries [40], but the negative impacts on male fertility were unable to be identified. However, homozygous splicing site variants (c.G2783A) leading to an abnormal RNA splicing site have been reported. Overexpression of the mutated *BRDT* gene can result in gain-of-function changes in the *BRDT* protein [26] and is associated with headless defects in human sperm. It may be assumed that the BRDT protein is involved in a functional distal centriole, elongating the sperm tail. As emphasised by Toyama et al. [7], Chemes et al. [9], and Li et al. [26], the intact mitochondrial sheath was not present in ultrastructural observation of *BRDT* mutation-associated AS sperms.

## 3. The Outcomes of ICSI Can Be Predicted by AS Subtypes

For abnormal sperm such as in the case of teratozoospermia, the lower cutoff value for morphology is an indication for an ICSI technique [5]. It overcomes the inability of sperm to undergo capacitation, move towards the oocyte, participate in the acrosome reaction, and cross the zona pellucida. This technique can solve the problems of sperm defects, such as a low sperm concentration, abnormal morphology, and inadequate motility [5]. Elkhatib et al. [42] pointed out that assisted reproductive technology, such as ICSI methods, provides a therapeutic solution for almost all infertile couples with AS defects. Based on an ICSI protocol, sperm showing an intact morphology may be chosen to fertilise eggs, in case genetic material may be lost during head-tail separation [13, 43]. Therefore, in the most reported AS cases, an abnormal head-tail junction sperm was used during an ICSI procedure instead of a tailless head of sperm alone.

Zhu et al. [2, 10] stressed that good quality embryos can be obtained by using head-tail sperm with *SUN5* or *PMFBP1* mutated genes. Clinical pregnancies were achieved after good quality embryos were transferred into women (Table 2). In addition, the same outcome also occurs with the mutated *HOOK1* gene that is related to headless defects. Healthy children were born that carried one of the AS associated mutated genes inherited from the paternal side [12]. Despite abnormal sperm morphology in subtype I and the good quality of embryos, several transferred embryos still failed to develop and pregnancy was not attained; implantation failure may be another possible cause [23]. The embryo implantation is an essential process that only half the embryos can be implanted successfully to achieve a sustained pregnancy [44]. However, ICSI achievements using head-tail connected sperm in human studies with mutated *SUN5*, *HOOK1*, and *PMFBP1* genes have shown these only affected the morphology of the sperm [2, 10, 12, 27]. It can be demonstrated that clinical pregnancy can be achieved in the case of acephalic spermatozoa, caused by *SUN5*, *HOOK1*, and *PMFBP1* gene variations.

By contrast, the ICSI results for subtypes I and III were unexpected. Chemes et al. [9] highlighted that syngamy and cleavage could not occur in a fertilised embryo using the tailless head of spermatozoa in subtype I since the distal centriole was missing from the sperm head. Meanwhile, in the case of *TSGA10* gene-related AS defects, a tailless head was able to fertilise an egg, but embryo quality was poor and clinical pregnancies failed to occur. Microscopic observation showed that due to irregular cleavage and developmental delay in the embryo, a clinical pregnancy failed after embryo transfer [45]. Meanwhile, the ICSI outcomes associated with *BRDT* mutation-associated AS defects have not been published as yet; however, the possible ICSI results of AS patients with a mutated *BRDT* gene can be predicted due to the defect of the distal centriole. In addition to the sperm centrosome, Ge et al. [46] and Miao et al. [47] described in detail how the poor quality of oocyte affects the development of embryos, such as ageing oocyte, dysfunction of mitochondria, and embryo implantation. However, the experimental studies in both human and animals confirmed that the poor embryo quality obtained through ICSI methods using the sperm from subgroups I and III and subsequent failure of clinical pregnancies and the lack of a distal centriole in subtype I and centriole defects due to subtype III genes were apparent [9, 26] (shown in Figure 1). It is suggested that the dysfunction of centrosome should be responded to an arrested fertilized oocyte [48]. Interestingly, Gambera et al. [49] demonstrated that a donor centrosome may be an alternative solution for AS patients with centriolar defects to avoid development failure in the embryo.

Both the unique morphology and genetic variation of AS sperm subtypes may affect the development failure in embryos (shown in Table 2 and Figure 1): the complete centrosome/centrioles of the sperm are important for embryonic development [24, 50]. Galotto et al. [51] and Hart [44] stressed the essential role of sperm centrioles in the early development of the embryo, such as migration of pronuclei, completion of the fertilization, and stimulating the first cleavage of zygote. The development of the embryo requires two centrioles [52]. Porcu et al. [23] believed that the lack of or defect of sperm centrioles may result in a cleavage failure of zygote and/or abnormal development of the embryo. For example, the embryo was observed to become arrested at the four-cell stage when using tailless sperms with a defect centriole [12]. Both the genetic origin and unique morphology of sperm with AS defects affect embryological outcomes [52, 53]. Therefore, identifying genetic variations and subtypes of AS defects may be critical, as well as evaluating risk information for infertile couples by using ICSI methods and their modifications.

Undoubtedly, the ICSI and clinical pregnancy achievements depend on not only normal morphological and functional sperm centrioles but also normal functional oocytes as well. A successful reproduction of the ICSI procedure cannot focus on the function and morphology of sperm alone. However, the failure of ICSI results of AS defects suggested that a dysfunctional centrosome was responsible for an arrested fertilised oocyte. Regarding the importance of paternal centrioles during embryonic development, possible mechanisms of pregnancy failure in I and III subtypes of AS sperm have been discussed in depth. Therefore, both the embryonic developments and the possible ICSI results of AS defects can be predicted: sperm that match the description of subgroup II can achieve better ICSI outcomes than sperm from subtypes I and III, which are unable to achieve oocyte development and clinical pregnancy. In conclusion, with regard to ICSI, outcomes of AS defects are limited by the small number of both human and animal studies and still need further research, while the existence of implantation failure in women cannot be ignored.

## Figures and Tables

**Table 1:** The classification of AS defects based on ultrastructural observations, broken points of the neck region of sperm, and AS target genes. AS: acephalic spermatozoa.

Subtypes	Separation point	Phenotype	Candidate gene	References
I	Between the two centrioles	The tailless head contains the complete proximal centriole with intact implantation fossa and basal body		Holstein et al. [20]

II	Between the nucleus and proximal centriole	The sperm head contains the intact nucleus and acrosome with absent basal body and/or aplasia of the implantation fossa	*HOOK1*,	Chemes and Rawe [8]
			*SUN5*,	Zhu et al. [10]
			*PMFBP1*	Chen et al. [12]

III	Between the distal centriole and mitochondrial sheath	The sperm tail can be observed with/without the incomplete mitochondrial sheath	*TSGA10*	Toyama et al. [7]
			*BRDT*,	Li et al. [26]
				Sha et al. [11]

**Table 2:** Mutation table of AS defects with reported intracytoplasmic sperm injection (ICSI) outcomes.

Gene	Mutation (HGVS nomenclature)	Mutation type	ICSI outcomes (sperm type)	Reference
*SUN5*	c.211 + 1 insGT^※^	Frameshift	Good quality embryos, clinical pregnancy was obtained, live births (head-tail still joined sperm)	Zhu et al. [2]
	c.216G > A^※^	Nonsense		
	c.340G > A^※^	Splicing		Shang et al. [33]
	c.381delA^※^	Frameshift		Shang et al. [27]
	c.425+1G > A^※^	Splicing		
	c.475C > T^※^	Nonsense		
	c.485T > A^※^	Missense		
	c.781G > A^※^	Missense		
	c.824C > T^※^	Missense		
	c.829C > T^※^	Nonsense		
	c.851C > G^※^	Nonsense		
	c.1043A > T^※^	Missense		
	c.1066C > T^※^	Missense		
	p.(Leu143Serfs^∗^30)^※^	Frameshift		
	c.1067G > A^※^	Missense		

*PMFBP1*	c.327T > A^※^	Nonsense	Good quality embryos, clinical pregnancy was obtained, live births (head-tail sperm)	Sha et al. [11]
	c.1462C > T^※^	Nonsense		Zhu et al. [10]
	c.2092delG^※^	Frameshift		
	c.2404C > T^※^	Nonsense		
	c.2561_2562del^※^	Frameshift		
	c.2725C > T^※^	Nonsense		

*TSGA10*	c.211del^※^	Frameshift	Poor quality embryo and failed clinical pregnancy (NG)	Sha et al. [34]

*BRDT*	c.G2783A^#^	Splicing	NG	Li et al. [26]

*HOOK1*	p.Q286R^※^	Nonsense	Clinical pregnancy was obtained, live births in both animal and human studies (NG)	Chen et al. [12]

HGVS: human genome variation society. NG: not given. AS: acephalic spermatozoa. ^#^Gain-of-function. ^※^Loss-of-function.
